# Angiopoietin-like-4 and minimal change disease

**DOI:** 10.1371/journal.pone.0176198

**Published:** 2017-04-25

**Authors:** Gabriel Cara-Fuentes, Alfons Segarra, Cecilia Silva-Sanchez, Heiman Wang, Miguel A. Lanaspa, Richard J. Johnson, Eduardo H. Garin

**Affiliations:** 1 Division of Pediatric Nephrology, Department of Pediatrics, University of Florida, Gainesville, Florida, United States of America; 2 Division of Nephrology, Hospital Vall d'Hebron, Barcelona, Spain; 3 Proteomics and Mass Spectrometry, Interdisciplinary Center for Biotechnology Research, University of Florida, Gainesville, Florida, United States of America; 4 Division of Renal Diseases and Hypertension, Department of Medicine, University of Colorado, Denver, Colorado, United States of America; University of Houston, UNITED STATES

## Abstract

**Background:**

Minimal Change Disease (MCD) is the most common type of nephrotic syndrome in children. Angiopoietin-like-4 (Angplt4) has been proposed as mediator of proteinuria in MCD. The aim of this study was to evaluate the role of Angptl4 as a biomarker in MCD.

**Methods:**

Patients with biopsy-proven primary MCD, focal segmental glomerulosclerosis, membranous nephropathy (60, 52 and 52 respectively) and 18 control subjects had urinary and serum Angptl4 measured by Elisa. Frozen kidney tissue sections were stained for Angptl4.

**Results:**

Angptl4 was not identified in glomeruli of MCD patients in relapse. Urinary Angptl4 levels were elevated in MCD in relapse as well as in patients with massive proteinuria due to other glomerular diseases.

**Conclusion:**

Neither serum nor urine Angptl4 appear to be good biomarkers in MCD. Elevated urinary Angptl4 n glomerular disease appears to reflect the degree of proteinuria rather than any specific disease.

## Introduction

Nephrotic syndrome is defined by the presence of massive proteinuria, hypoalbuminemia, edema and hypercholesterolemia. Minimal Change Disease (MCD), the most common type of nephrotic syndrome in children [1], is thought to be triggered by a circulating factor (s) yet identified [2]. This factor is posited to induce changes in podocytes leading to increased glomerular permeability to plasma proteins. Historically products from inflammatory cells such as interleukins were presumed to trigger MCD, although firm evidence of this pathway is still lacking despite more than 40 years of research [3].

Human Angiopoietin-like-4 (Angptl4) is a 45–65 kDa glycoprotein highly expressed in liver and adipose tissue. Podocyte Angtpl4 has been proposed to have a role in the development of proteinuria in MCD [4–6]. Angptl4 podocyte overexpression has been reported in MCD in relapse [4] and in other human glomerular diseases [7–9].

The aim of this study is to evaluate the role of Angptl4 as a biomarker in MCD.

## Methods

### Definitions

All patients had either biopsy-proven primary MCD, focal segmental glomerulosclerosis (FSGS) or membranous nephropathy (MN) [10, 11]. Relapse was defined as the presence of proteinuria (urinary protein creatinine ratio > 2.0 mg/mg or 3 + or greater by using the tetrabromophenol-citrate buffer colorimetric qualitative test—dipstick- or >3 grams of protein in 24 hour urine collection), serum albumin ≤3.5 g/d, and edema. Complete remission was defined as negative proteinuria by dipstick or urinary protein creatinine ratio <0.2 mg/mg. Patients with urinary protein creatinine ratio greater than 0.2 but lower than 2 were included in the remission group as they were considered in early remission based on subsequent clinical course.

### Patients

164 patients (18 children and 146 adults) with biopsy proven MCD, FSGS or MN and 18 control subjects were included in this study (**see** Table 1 **and** S1–S4 Tables for details). Sixty patients had MCD (18 children and 42 adults) of which 44 were studied during relapse and 26 during remission. Ten patients were studied during both relapse and remission. Fifty-two patients had primary FSGS as their underlying glomerular disease. Thirty-six and sixteen patients were studied in relapse and in remission respectively. Of the 52 patients with primary MN, 36 were studied during relapse and 16 during remission. Eighteen subjects served as controls. These individuals had essential hypertension (n = 8), microscopic hematuria (n = 2), nephrogenic diabetes insipidus (n = 2), urinary tract infection (n = 1), solitary kidney (n = 1), urinary incontinence (n = 1), simple kidney cyst (n = 1), hypoaldosteronism (n = 1) and enuresis (n = 1).

**Table 1:** Demographic characteristics of patients and control subjects.

Groups→	MCD^a^ (n 60)		FSGS^b^ (n 52)		MN^c^ (n 52)		Control (n 18)
Variable	Relapse	Remission	Relapse	Remission	Relapse	Remission	
**Patients (n)**^d^	44	26	36	16	36	16	18
**Age (years)**	30.7±20.2	20.2±16.1	48±12.7	44.1±12.2	54.1±13	51.4±9.1	13.3±3.9
**Gender** **(male/female—n-)**	26/18	14/12	28/8	12/4	23/13	10/6	12/6
**Serum albumin (g/dl)**	2.2±0.5	4.1±0.3 (n 22)	2.9±0.6	3.8±0.4	2.5±0.5	3.3±0.2 (n 3)	4.7±0.2 (n 7)
**Proteinuria (g/24h)**	12±3.5 (n 29)	NA^e^	6.6±3.5 (n 21)	NA	7.4±3.1 (n 20)	NA	NA
**Urine Protein to creatinine ratio (mg/mg)**	7.4±5.4 (n 14)	0.3±0.4 (n 23)	6.6±3.4 (n 15)	0.7±0.6	6.4±4.7 (n 16)	0.8±0.6	0.06±0.03 (n 9)
**Urine protein by dipstick**	>300 mg/dl (n 1)	Negative (n 3)	NA	NA	NA	NA	Negative (n 9)
**Serum creatinine (mg/dl)**	0.7±0.2 (n 40)	0.6±0.3 (n 22)	1.1±0.3	1.4±0.7 (n 15)	1±0.3	1±0.2 (n 15)	0.6±1.9 (n 13)

^a^MCD minimal change disease,

^b^FSGS focal segmental glomerulosclerosis,

^c^MN membranous nephropathy,

^d^n refers to the number of patients,

^e^NA not available, data expressed as mean±SD.

Children and adult subjects were followed at the University of Florida and at Vall d’Hebron Hospital in Barcelona (Spain) respectively. The study was performed according to the Declaration of Helsinki and approved by the Institutional Review Board of the University of Florida (IRB protocol#481–2008) and Vall d’Hebron Hospital [(AC/I(AG)158/2013(3822)]. Written informed consents were obtained from all participants and from caretakers/guardians of minors. Written assents were also obtained when indicated.

### Serum and urine collection

Blood samples from patients and control subjects were centrifuged for 5 minutes at 2400 RPM and serum collected and stored at −80°C. Urine samples from the above mentioned patients and controls were also stored at -80°C until samples were analyzed.

### Serum and urinary Angptl4

Angplt4 levels were measured using a commercially available Enzyme-Linked Immunosorbent Assay (ELISA) (Sigma-Aldrich, catalog number RAB0017). Serum Angplt4 levels were expressed as ng/ml and urinary Angptl4 as ng/g of creatinine.

### Serum albumin and urine protein and creatinine

Proteinuria, measured by autoanalyzer, was expressed as grams of protein in 24 hour urine collection and as protein to creatinine ratio in random urine samples.

### Immunohistochemistry of Angptl4 of human kidney tissue specimens

Cryosections of kidney biopsy from patients were fixed with pre-cooled ice old acetone for 10 min. The sections were wash twice with PBS and then incubated with block solution (5% goat serum and 5% donkey serum in PBS) for 30 min. Afterwards, the sections were incubated with anti-synaptopodin antibody (American Research Products, INC) at room temperature for 1 hour, followed by rabbit polyclonal anti-Angptl4 antibody (Catalog number#sc-66806, Santa Cruz, 1:50) for 1 hour. After 3 times of PBS wash, each for 5 minutes, the sections were incubated with chicken anti-mouse 488 and chicken anti-rabbit 594 Alexa Fluor secondary antibodies (Thermo Fisher, 1:1000) for 1 hour. After 3 times of PBS wash, 5 minutes each, the sections were incubated with DAPI for counterstaining. The images were then taken and analyzed with confocal microscopy (Leica). Clinical data of patients whose renal tissue was stained are provided in S5 Table.

### Statistical analysis

Statistical analysis was performed using the statistical software GraphPad Prism 6. The non-parametrical tests, Kruskal–Wallis and Mann–Whitney U, were applied to evaluate differences between the groups. Spearman and Pearson correlation coefficient were calculated to determine statistical correlation between two numerical variables as indicated. *P* < 0.05 was regarded as statistically significant. Data were expressed as mean ± standard deviation (SD) for normally distributed data and median +/- interquartile 25%-75% (IQ) when data were not normally distributed.

## Results

Demographics and laboratory tests are shown on Table 1 (see S1–S4 Tables for details).

### Urinary and serum Angptl4

**Fig 1:**
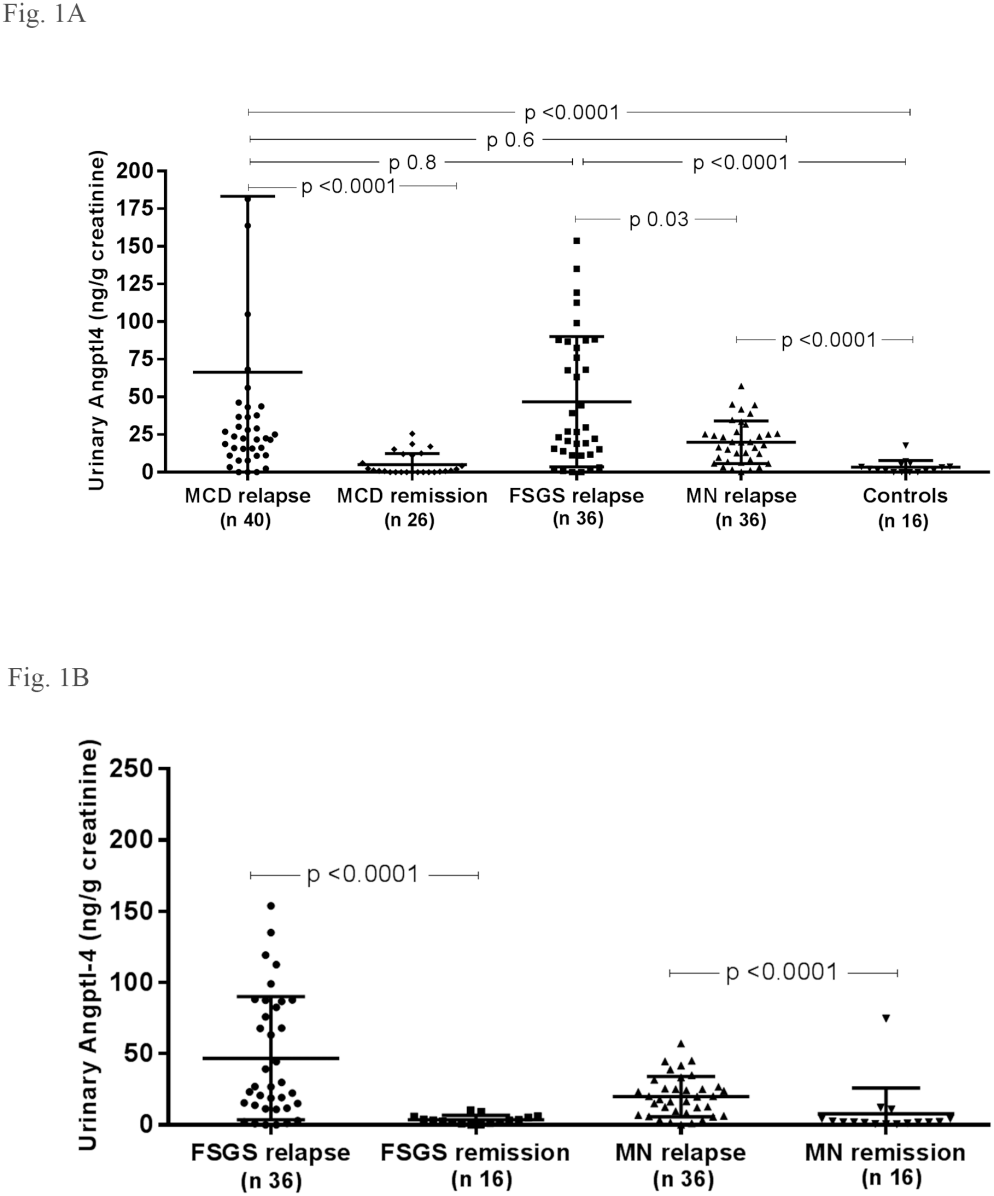
Urinary Angptl4 in MCD, FSGS, MN patients and control subjects. (A) Urinary Angptl4 in MCD, FSGS, MN patients and control subjects. (B) Urinary Angptl4 in FSGS and MN patients during relapse and remission. MCD minimal change disease, FSGS focal segmental glomerulosclerosis, MN membranous nephropathy, n refers to the number of patients, data expressed as mean±SD.

Urinary Angptl4 excretion was increased in patients with massive proteinuria regardless of underlying glomerular disease (MCD, FSGS and MN) compared to control subjects (p<0.0001 for each group (Fig 1A). Urinary Angptl4 excretion was significantly elevated in MCD (Fig 1A), FSGS and MN patients (Fig 1B) during relapse compared to the same group of patients during remission (p< 0.0001 for each group) and to control subjects.

**Fig 2:**
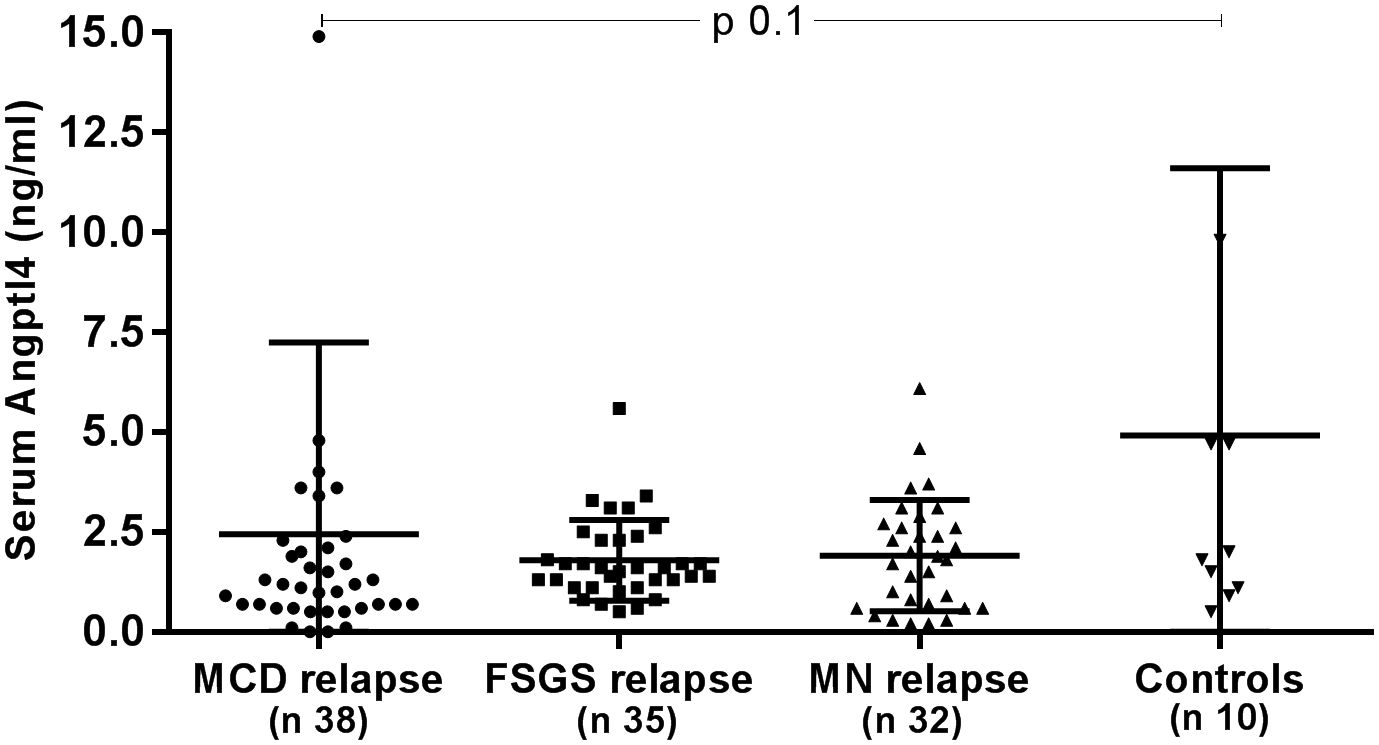
Serum Angptl4 in MCD, FSGS, MN during relapse and control subjects. Angptl4 angiopoietin-like-4, MCD minimal change disease, FSGS focal segmental glomerulosclerosis, MN membranous nephropathy, n refers to the number of patients, data expressed as mean±SD.

Serum Angptl4 levels were decreased in MCD, FSGS and MN patients in relapse compared to control subjects, although this difference did not reach statistical significance (Fig 2).

No statistical differences were observed in serum Angptl4 levels among patients with MCD, FSGS and MN patients during relapse compared to the same group of patients during remission and in those patients during remission compared to controls (S1A and S1B Fig **respectively**).

There was a positive correlation between urinary and serum Angptl4 levels in MN patients in relapse (p 0.02) but not in the MCD and FSGS groups during relapse (S2A–S2C Fig).

#### Angptl4, serum albumin and proteinuria

During relapse, patients with MCD had a higher level of proteinuria compared to FSGS and MN patients (p<0.0001) (S3A Fig). The degree of proteinuria was not statistically different among patients with FSGS and MN during relapse (S3A Fig). There was a negative correlation between proteinuria and serum albumin levels in MCD and MN patients during relapse (p<0.0001 and p 0.001 respectively) (S3B and S3C Fig **respectively**). This correlation did not reach statistical significance in the FSGS group (S3D Fig).

Urinary Angptl4 excretion in relapse correlated with proteinuria in the FSGS and MN groups. This positive correlation was not found for MCD patients during relapse (S4 Fig). No correlation was found between serum Angplt4 levels and proteinuria in MCD, FSGS and MN patients during relapse (S5 Fig).

#### Glomerular Angptl4 expression

**Fig 3:**
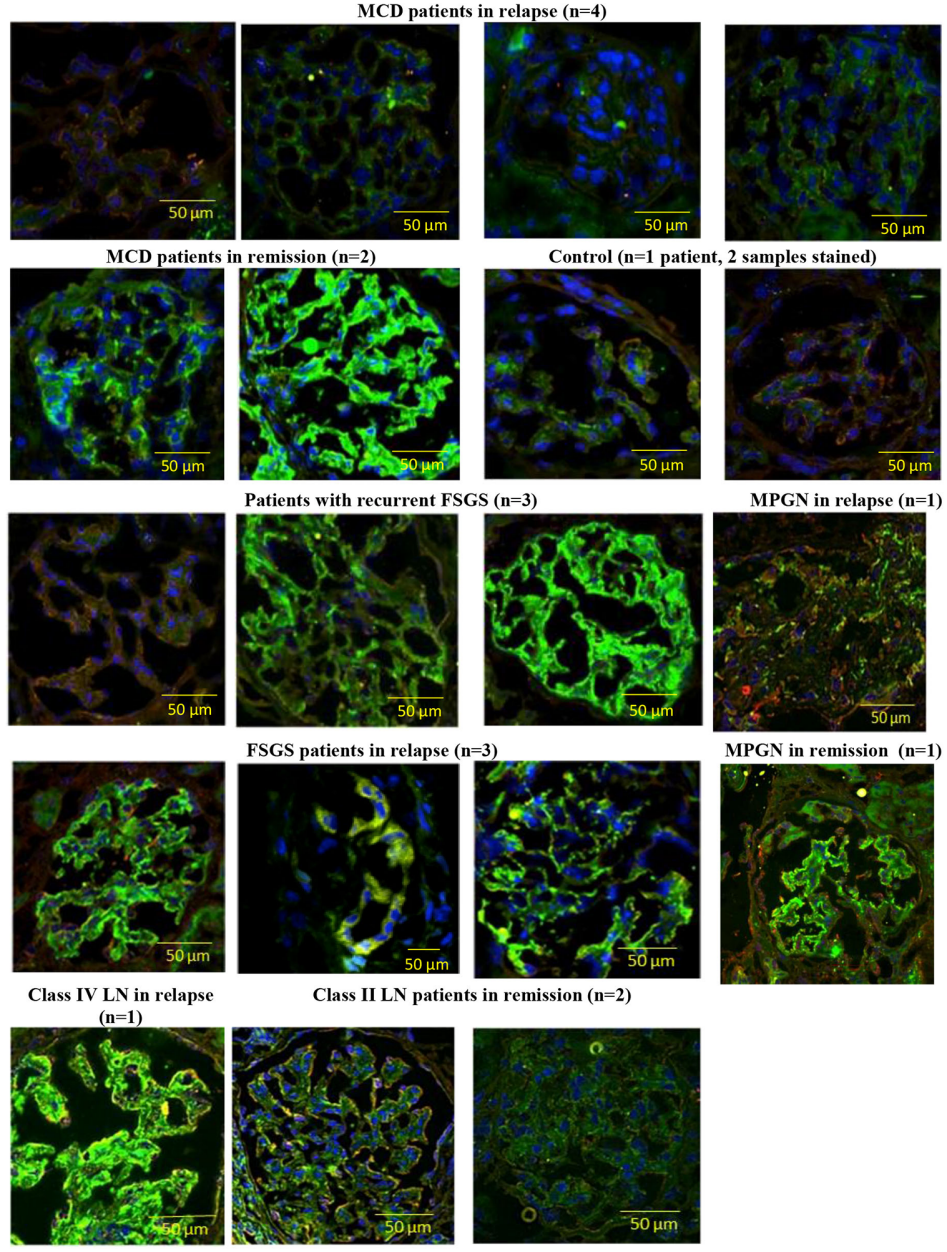
Glomerular Angplt4 staining in patients with MCD and other glomerulopathies. Angptl4 angiopoietin-like-4, MCD minimal change disease, FSGS focal segmental glomerulosclerosis, LN lupus nephritis, MPGN membranoproliferative glomerulonephritis, n refers to the number of patients, Green, blue and red stains represent Angptl4, DAPI and synaptopodin respectively. Yellow lines represent scale bars. Magnification x200.

Angptl4 staining was absent (as in control) in glomeruli from 4 MCD patients in relapse, 1 patient with membranoproliferative glomerulonephritis (MPGN) in relapse, and 1 of 6 with FSGS and heavy proteinuria (Fig 3).

Two patients with class II lupus nephritis in remission showed minimal staining for glomerular Angptl4. Compared to controls, glomerular Angptl4 was overexpressed in kidney tissue from 2 MCD patients in remission, 5 patients with FSGS and proteinuria, 1 patient with class IV lupus nephritis during relapse and 1 MPGN patient during remission.

## Discussion

We evaluated Angptl4 as a biomarker in MCD. The primary finding was that the serum and urinary excretion of Angptl4 were not specific for MCD, and there was no correlation of either serum or urine Angptl4 with proteinuria in MCD patients. Glomerular Angptl4 immunohistochemistry was negative in control subjects, variably expressed in subjects with MCD in remission, FSGS and MN, and absent in MCD subjects in relapse. Thus, this study could not document urine or serum Angptl- 4 to be a biomarker of MCD.

One group previously reported increased glomerular Angptl4 by immunohistochemistry in 5 patients with MCD compared to controls, all from kidneys prior to transplantation [4]. Li et al also reported glomerular Angplt4 expression, by immunostaining, in a non-specified number of MCD and MN patients during active nephrotic syndrome, but they did not include normal controls or MCD patients in remission [7]. Our study, however, was unable to confirm these findings in MCD, and instead found increased expression of Angptl4 in 2 MCD patients during remission, in 5 of 6 patients with active FSGS, in 1 case of MPGN in relapse, and 1 subject with class IV lupus nephritis during relapse and in 1 MPGN patient with mild proteinuria (Fig 3). It is possible these differences reflect the fact that different primary antibodies to Angptl- 4 were used although each was raised against Angptl-4 containing the N-terminus domain. Our study used a rabbit polyclonal antibody from Santa Cruz, while Li used a primary goat antibody from Santa Cruz, and Chugh used an antibody raised in his own laboratory, S6 Table). Unfortunately, the antibody used by Chugh is no longer available, thus preventing cross-testing. D’Agati has noted, regarding CD80 glomerular staining, another controversial subject, that immunohistochemistry is a tricky technique subject to numerous variables including “quantity of antigen, type and duration of fixation and image exposure time and contrast” [12]. Thus, whether Angptl4 is expressed in MCD patients during relapse requires further study.

Elevated plasma Angptl4 levels has been reported in patients with nephrotic syndrome due to MCD, FSGS, MN and crescentic glomerulonephritis compared to control subjects [5]. In contrast, we found lower serum Angplt4 levels in patients with MCD, FSGS and MN during relapse compared to controls. While we used a different ELISA kit compared to that used by Chugh, both kits used goat antihuman polyclonal antibodies raised against the same amino acids (aa) (aa 26–406) (S7 Table) and should recognize the full Angptl-4 molecule. Furthermore, the mean plasma Angptl4 reported by Chugh et al in control subjects is 2–10 times higher than that published by other authors using a similar assay [13–17]. The mean serum levels of normal control subjects observed in our study is similar to that described by these other authors (S7 Table) [13–17].

We documented marked elevations in urine Angptl4 in patients with massive proteinuria regardless of the underlying disease (MCD, FSGS and MN) compared to the same group of patients during remission and to normal controls. Li et al reported higher urinary Angptl4 levels in MCD in relapse compared to five patients with mesangial proliferative glomerulonephritis (mPGN) of which two of the latter had absent or mild proteinuria [7]. Li et al also reported an increase in urinary Angptl4 expression, by Western blot, in patients with MCD, FSGS and MN with proteinuria compared to that observed in mPGN patients, also consistent with a relationship of Angptl4 urinary excretion to proteinuria rather than to MCD [7]. These data are consistent with urinary Angptl4 being a biomarker of proteinuria. Indeed, the molecular weight of circulating Angptl4 is 50 kDa and would be expected to be excreted in the urine similar to other small molecular weight proteins.

Chugh’s group has presented experimental evidence supporting a role for podocyte Angptl4 as a mediator of proteinuria in MCD and has suggested that it acts by altering the charge barrier of the glomerular capillary wall. Our data do not prove or disprove this hypothesis, although we would suggest that the variable expression of Angptl4 that we observed in glomeruli may reflect nonspecific binding of Angptl4 to glomerular structures, such as binding to extracellular matrix [18] [19] or endothelial cells [20][5]. It is also possible that the antibody we used for immunofluorescence could also be binding to fibrinogen since a fibrinogen domain is present in the carboxy terminal of the Angptl4 molecule. However, the antibody we used was produced against the N-terminus domain of the molecule, and this would also be unlikely to explain the positive staining of glomeruli of MCD subjects in remission.

In summary, we could not confirm the presence of Angptl4 in glomeruli of MCD patients in relapse. Elevated urinary Angptl4 is not a specific marker for MCD and more likely reflects nephrotic range proteinuria.

## Supporting information

S1 Fig (A) Serum Angptl4 in MCD, FSGS and MN patients during relapse and remission and (B) in MCD, FSGS and MN patients during remission and control subjects. Angptl4 angiopoietin-like-4, MCD minimal change disease, FSGS focal segmental glomerulosclerosis, MN membranous nephropathy, n refers to the number of patients, data expressed as mean±SD.
(DOC)

S2 Fig Correlation between serum and urinary Angplt4 in (A) MCD, (B) FSGS and (C) MN patients in relapse. Angptl4 angiopoietin-like-4, MCD minimal change disease, FSGS focal segmental glomerulosclerosis, MN membranous nephropathy, n refers to the number of patients.
(DOC)

S3 Fig (A) Degree of proteinuria (showed as grams/24 hours) according to glomerular disease and correlation between serum albumin and degree of proteinuria (showed as grams/24 hours) in (B) MCD, (C) MN and (D) FSGS patients during relapse. MCD minimal change disease, FSGS focal segmental glomerulosclerosis, MN membranous nephropathy, n refers to the number of patients.
(DOC)

S4 Fig Correlation between proteinuria (showed as urine protein to creatinine ratio) and urinary Angptl4 in (A) MCD, (B) FSGS and (C) MN patients during relapse. Angptl4 angiopoietin-like-4, MCD minimal change disease, FSGS focal segmental glomerulosclerosis, MN membranous nephropathy, n refers to the number of patients.
(DOC)

S5 Fig Correlation between proteinuria (showed as urine protein to creatinine ratio) and serum Angptl4 in (A) MCD, (B) FSGS and (C) MN patients during relapse. Angptl4 angiopoietin-like-4, MCD minimal change disease, FSGS focal segmental glomerulosclerosis, MN membranous nephropathy, n refers to the number of patients.
(DOC)

S1 Table Demographic and laboratory data from patients with MCD in (A) relapse and (B) remission. MCD minimal change disease, UPC urine protein to creatinine ratio, Angptl4 angiopoietin-like-4, F female, M male, NA not available, * excluded from mean as it represents protein detected by dipstick, Negative refers to negative protein by dipstick, SD standard deviations, IQ interquartile 25–75% percentile, data presented as mean±SD and median (IQ) when data were not normally distributed, † non normally distributed data.
(DOC)

S2 Table Demographic and laboratory data from patients with FSGS in (A) relapse and (B) remission. FSGS focal segmental glomerulosclerosis, UPC urine protein to creatinine ratio, Angptl4 angiopoietin-like-4, F female, M male, NA not available, SD standard deviations, IQ interquartile 25–75% percentile, data presented as mean±SD and median (IQ) when data were not normally distributed, † non normally distributed data.
(DOC)

S3 Table Demographic and laboratory data from patients with MN in (A) relapse and (B) remission. MN membranous nephropathy, UPC urine protein to creatinine ratio, Angptl4 angiopoietin-like-4, F female, M male, NA not available, SD standard deviations, IQ interquartile 25–75% percentile, data presented as mean±SD and median (IQ) when data were not normally distributed, † non normally distributed data.
(DOC)

S4 Table Demographic and laboratory data from control subjects. UPC urine protein to creatinine ratio, Angptl4 angiopoietin-like-4, F female, M male, NA not available, SD standard deviations, IQ interquartile 25–75% percentile, negative refers to negative protein by dipstick, data presented as mean±SD and median (IQ) when data were not normally distributed, † non normally distributed data.
(DOC)

S5 Table Demographic and laboratory data from patients and control whose kidney tissue was stained for Angplt4. Angptl4 angiopoietin-like-4, MCD minimal change disease, FSGS focal segmental glomerulosclerosis, MPGN membranoproliferative glomerulonephritis, * individuals not included in any of the studied groups. Data from these individuals were not included in Table 1 nor figures, UPC urine protein to creatinine ratio, ** Negative refers to negative protein by dipstick.
(DOC)

S6 Table Angptl4 antibodies used for staining human kidney tissue. Angptl4 angiopoietin-like-4, MW molecular weight, kDa kilodalton, aa aminoacids.
(DOC)

S7 Table Summary of Angptl4 levels in plasma/serum from humans and characteristic of antibodies used for Angptl4 quantification. Angptl4 angiopoietin-like-4, aa aminoacids, y year-old, N refers to the number of patients, SD standard deviation, SEM standard error of mean, IQ interquartile, * “Elisa assay virtually identical to the Duoset Elisa Angptl4 offered commercially by R&D (DY3485)”.
(DOC)
