# Supplementary material for: Angiopoietin-like-4 and minimal change disease
Source: PLoS One. 2017 Apr 25;12(4):e0176198. doi: 10.1371/journal.pone.0176198 (PMC5404758; doi:10.1371/journal.pone.0176198)
Supplement: S1 Fig — Angptl4 angiopoietin-like-4, MCD minimal change disease, FSGS focal segmental glomerulosclerosis, MN membranous nephropathy, n refers to the number of patients, data expressed as mean±SD. (DOC) [file pone.0176198.s001.doc]

**S1 Fig.**

a)

b)
