# Supplementary material for: Angiopoietin-like-4 and minimal change disease
Source: PLoS One. 2017 Apr 25;12(4):e0176198. doi: 10.1371/journal.pone.0176198 (PMC5404758; doi:10.1371/journal.pone.0176198)
Supplement: S3 Fig — MCD minimal change disease, FSGS focal segmental glomerulosclerosis, MN membranous nephropathy, n refers to the number of patients. (DOC) [file pone.0176198.s003.doc]

**S3 Fig.**

a) b)

c) d)
