# Supplementary material for: Angiopoietin-like-4 and minimal change disease
Source: PLoS One. 2017 Apr 25;12(4):e0176198. doi: 10.1371/journal.pone.0176198 (PMC5404758; doi:10.1371/journal.pone.0176198)
Supplement: S1 Table — MCD minimal change disease, UPC urine protein to creatinine ratio, Angptl4 angiopoietin-like-4, F female, M male, NA not available, * excluded from mean as it represents protein detected by dipstick, Negative refers to negative protein by dipstick, SD standard deviations, IQ interquartile 25–75% percentile, data presented as mean±SD and median (IQ) when data were not normally distributed, † non normally distributed data. (DOC) [file pone.0176198.s006.doc]

**S1 Table**.

| **Table 1A. MCD patients in relapse (n=44, 13 children, 31 adults)** | | | | | | | | |
| --- | --- | --- | --- | --- | --- | --- | --- | --- |
| **Patient** | **Gender** | **Age (years)** | **Serum albumin (g/dl)** | **Proteinuria** | | **Urinary Angptl4**  **(ng/g creatinine)** | **Serum Angptl4**  **(ng/ml)** | **Serum creatinine**  **(mg/dl)** |
|  |  |  |  | **UPC** | **Grams/24h** |  |  |  |
| 1 | F | 7 | 2.5 | 13.7 | NA | 46.2 | 1.7 | 0.3 |
| 2 | M | 3 | 2 | 4.4 | NA | 43.2 | 3.4 | 0.2 |
| 3 | F | 10 | 1.4 | 12.6 | NA | 181.4 | 0.7 | 0.4 |
| 4 | M | 10 | 1.9 | 4.2 | NA | 615.7 | 14.9 | 0.5 |
| 5 | M | 8 | 1.8 | 20.4 | NA | 36.7 | 1.9 | 0.6 |
| 6 | F | 4 | 1.7 | 5.3 | NA | 11.2 | NA | NA |
| 7 | F | 14 | 1.9 | 4.3 | NA | 288.6 | 4.8 | 0.5 |
| 8 | M | 5 | 1.9 | 12.9 | NA | 2.4 | 27.1 | 0.3 |
| 9 | M | 3 | 3.5 | 4.4 | NA | 0 | NA | 0.3 |
| 10 | F | 11 | 2.6 | 3.3 | NA | 21.8 | 0.7 | 0.4 |
| 11 | M | 19 | 3.9 | > 300* | NA | 0 | NA | 0.5 |
| 12 | M | 5 | 3.1 | 4.3 | NA | 28 | 2.1 | 0.3 |
| 13 | M | 8 | 3.9 | 1.1 | NA | 0 | NA | NA |
| 14 | M | 55 | 2.1 | 8.3 | NA | 56.1 | NA | NA |
| 15 | F | 32 | 2.6 | 5.3 | NA | 15.6 | NA | NA |
| 16 | M | 21 | 2.3 | NA | 10.2 | 68.3 | 0.9 | 0.9 |
| 17 | M | 34 | 2.2 | NA | 11 | 36.5 | 1 | 1.2 |
| 18 | F | 21 | 1.9 | NA | 9.2 | 22.6 | 0.5 | 0.78 |
| 19 | M | 42 | 1.7 | NA | 14 | 16.2 | 0.1 | 0.57 |
| 20 | F | 31 | 2.4 | NA | 15 | 293.4 | 3.6 | 0.83 |
| 21 | M | 76 | 2.3 | NA | 12 | 23.7 | 0 | 0.7 |
| 22 | M | 26 | 2.4 | NA | 8.4 | 30.3 | 1.5 | 0.68 |
| 23 | F | 29 | 1.4 | NA | 16.3 | 29.0 | 0 | 0.9 |
| 24 | F | 35 | 3.1 | NA | 11 | 16 | 0.5 | 0.66 |
| 25 | F | 20 | 2.4 | NA | 8.7 | 21.6 | 2.4 | 0.9 |
| 26 | M | 35 | 2.3 | NA | 16 | 25 | 0.7 | 0.7 |
| 27 | F | 45 | 2.4 | NA | 15 | 22.4 | 2 | 0.8 |
| 28 | M | 45 | 1.9 | NA | 11.5 | 26.9 | 1.2 | 0.8 |
| 29 | M | 32 | 2.6 | NA | 7.5 | NA | 3.6 | 0.9 |
| 30 | M | 43 | 2.3 | NA | 7.9 | 252.7 | 2.3 | 1.02 |
| 31 | F | 75 | 1.8 | NA | 11.2 | NA | 1.6 | 0.76 |
| 32 | F | 28 | 2.9 | NA | 8.5 | 163.8 | 0.5 | 0.8 |
| 33 | F | 34 | 2.7 | NA | 9.4 | 104.9 | 0.6 | 1 |
| 34 | M | 33 | 2.2 | NA | 8.4 | 37.7 | 0.6 | 0.64 |
| 35 | M | 42 | 2.7 | NA | 6.7 | 43.8 | 0.1 | 0.86 |
| 36 | F | 55 | 2.4 | NA | 11.4 | 11.1 | 0.7 | 1 |
| 37 | M | 28 | 2 | NA | 12.6 | 7.7 | 0.7 | 1 |
| 38 | M | 72 | 1.8 | NA | 15.8 | 7.8 | 0.6 | 0.8 |
| 39 | F | 38 | 2.2 | NA | 13 | 15.8 | 1.2 | 0.9 |
| 40 | F | 27 | 2.8 | NA | 9.8 | 3.4 | 1.3 | 0.69 |
| 41 | M | 55 | 1.5 | NA | 14.5 | 18.8 | 1.1 | 1.53 |
| 42 | M | 19 | 1.4 | NA | 22 | NA | 4 | 0.9 |
| 43 | M | 45 | 2 | NA | 13 | NA | 0.9 | 0.8 |
| 44 | M | 72 | 1.5 | NA | 18 | 10.9 | 1.3 | 0.78 |
| Mean±SD |  | 30.7±20.2 | 2.2±0.5† | 7.4±5.4 | 12±3.5 | 66.4±116.9† | 2.4±4.7† | 0.7±0.2 |
| Median (IQ) |  |  | 2.2 (1.9-2.6) |  |  | 24.3 (12.3-45.6) | 1.1 (0.6-2.1) |  |

| **Table 1B. MCD patients in remission (n=26, 15 children, 11 adults)** | | | | | | | |
| --- | --- | --- | --- | --- | --- | --- | --- |
| **Patient** | **Gender** | **Age (years)** | **Serum albumin (g/dl)** | **UPC** | **Urinary Angptl4**  **(ng/g creatinine)** | **Serum Angptl4**  **(ng/ml)** | **Serum creatinine**  **(mg/dl)** |
| 1 | F | 6 | 4.5 | 0.1 | 2.4 | 19 | 0.3 |
| 2 | M | 3 | 3.5 | 0.2 | 0 | 1.3 | 0.3 |
| 3 | F | 9 | 4.7 | 0.08 | 0 | 0.6 | 0.4 |
| 4 | M | 10 | 4.5 | 0.1 | 0 | NA | 0.3 |
| 5 | M | 7 | 4.1 | 0.4 | 12 | 9.2 | 0.3 |
| 7 | F | 14 | 4.2 | 0.1 | 0.4 | 1.8 | 0.6 |
| 8 | M | 6 | 4.2 | Negative | 0 | 4.9 | 0.4 |
| 9 | M | 4 | NA | 0.1 | 2.1 | 6.2 | NA |
| 10 | F | 12 | 3.8 | 0.3 | 0.7 | 0.76 | 0.3 |
| 11 | M | 19 | 3.8 | 0.2 | 0 | 0.6 | 0.6 |
| 45 | F | 3 | 3.5 | 0.8 | 25.6 | 1.5 | 0.3 |
| 46 | F | 19 | NA | Negative | 0 | 3.3 | NA |
| 47 | F | 11 | NA | Negative | 0 | 0.3 | NA |
| 48 | M | 4 | 4.7 | 0.1 | 0 | 0.7 | 0.4 |
| 49 | M | 4 | NA | 0.08 | 0 | NA | NA |
| 50 | F | 38 | 4.5 | 1.1 | 17 | 1 | 0.9 |
| 51 | M | 28 | 4.1 | 1.1 | 12.6 | NA | 1 |
| 52 | F | 41 | 4.2 | 1.1 | 15.3 | NA | 0.9 |
| 53 | M | 55 | 4 | 0.08 | 18.7 | 0.4 | 1.5 |
| 54 | F | 45 | 4.2 | 0.09 | 4.3 | 1.2 | 0.8 |
| 55 | F | 27 | 4.1 | 0.9 | 0.9 | 1.3 | 0.6 |
| 56 | M | 19 | 4.2 | 0.03 | 1.3 | 0.8 | 0.9 |
| 57 | F | 18 | 4 | 0.03 | 6.1 | 0.03 | 0.7 |
| 58 | M | 46 | 4.2 | 0.2 | 1.1 | 0.8 | 0.8 |
| 59 | M | 44 | 4.3 | 0.003 | 11.2 | 9.4 | 0.9 |
| 60 | M | 35 | 4.1 | 1.1 | 0.7 | NA | 0.7 |
| Mean±SD |  | 20.2±16.1 | 4.1±0.3 | 0.3±0.4 | 5±7.3† | 3.1±4.5† | 0.6±0.3 |
| Median (IQ) |  |  |  |  | 1 (0-11.4) | 1.2 (0.6-4.1) |  |
