# Supplementary material for: Angiopoietin-like-4 and minimal change disease
Source: PLoS One. 2017 Apr 25;12(4):e0176198. doi: 10.1371/journal.pone.0176198 (PMC5404758; doi:10.1371/journal.pone.0176198)
Supplement: S2 Table — FSGS focal segmental glomerulosclerosis, UPC urine protein to creatinine ratio, Angptl4 angiopoietin-like-4, F female, M male, NA not available, SD standard deviations, IQ interquartile 25–75% percentile, data presented as mean±SD and median (IQ) when data were not normally distributed, † non normally distributed data. (DOC) [file pone.0176198.s007.doc]

**S2 table**.

| **Table 2A. FSGS patients in relapse (n=36)** | | | | | | | | |
| --- | --- | --- | --- | --- | --- | --- | --- | --- |
| **Patient** | **Gender** | **Age (years)** | **Serum albumin (g/dl)** | **Proteinuria** | | **Urinary Angptl4**  **(ng/g creatinine)** | **Serum Angptl4**  **(ng/ml)** | **Serum creatinine**  **(mg/dl)** |
|  |  |  |  | **UPC** | **Grams/24 h** |  |  |  |
| 1 | M | 35 | 2.8 | 9.2 | NA | 11 | 0.6 | 1.3 |
| 2 | M | 53 | 4.1 | 5.2 | NA | 88.2 | 3.1 | 2.5 |
| 3 | M | 59 | 2.9 | 3.3 | NA | 2.1 | 1.3 | 1 |
| 4 | M | 56 | 2 | 4.5 | NA | 22.3 | 1.1 | 0.9 |
| 5 | M | 61 | 2.9 | 7.9 | NA | 11.7 | 1.3 | 1.9 |
| 6 | F | 36 | 2.2 | 5.3 | NA | 0 | 0.8 | 1.6 |
| 7 | M | 45 | 2.9 | 11.7 | NA | 112.5 | 1.7 | 1.7 |
| 8 | F | 22 | 1.6 | 14 | NA | 86.8 | 2.4 | 0.6 |
| 9 | M | 68 | 3.2 | 8 | NA | 3 | 1 | 1.2 |
| 10 | M | 34 | 2.2 | 10.6 | NA | 20.8 | 0.7 | 1.2 |
| 11 | M | 43 | 3.4 | 4.2 | NA | 1.2 | 5.6 | 1.1 |
| 12 | M | 56 | 2.8 | 4.6 | NA | 44.4 | NA | 0.9 |
| 13 | F | 45 | 3 | 5.1 | NA | 67.8 | 0.5 | 1.3 |
| 14 | F | 57 | 3.1 | 2.8 | NA | 0.9 | 1.3 | 0.8 |
| 15 | M | 62 | 3.5 | 2.7 | NA | 0 | 1.6 | 1.3 |
| 16 | F | 46 | 2.9 | NA | 8 | 13.9 | 1.6 | 0.5 |
| 17 | F | 22 | 1.5 | NA | 18 | 11.3 | 2.3 | 0.6 |
| 18 | M | 68 | 3.2 | NA | 4 | 15.2 | 2.5 | 1.2 |
| 19 | M | 35 | 2.8 | NA | 11 | 18.9 | 3.1 | 1.3 |
| 20 | M | 53 | 4.15 | NA | 4.3 | 26.8 | 1.1 | 1.1 |
| 21 | M | 41 | 3.4 | NA | 4.4 | 19.3 | 2.6 | 1.2 |
| 22 | M | 49 | 3.5 | NA | 6.1 | 39.3 | 3.3 | 1 |
| 23 | M | 23 | 3.8 | NA | 8.2 | 27 | 2.3 | 1.2 |
| 24 | M | 41 | 3 | NA | 4 | 23.2 | 1.7 | 1.1 |
| 25 | M | 40 | 3.3 | NA | 4.8 | 15.6 | 1.7 | 1.2 |
| 26 | M | 39 | 3.1 | NA | 4 | 68 | 0.8 | 1.2 |
| 27 | M | 57 | 3.6 | NA | 6.9 | 153.8 | 1.6 | 1.1 |
| 28 | M | 62 | 3.5 | NA | 4.3 | 135.1 | 1.7 | 1.3 |
| 29 | M | 59 | 2.9 | NA | 6 | 87.9 | 1.5 | 1 |
| 30 | M | 60 | 1.9 | NA | 8.1 | 63.2 | 1.4 | 1.2 |
| 31 | M | 56 | 2 | NA | 7.1 | 82.6 | 1.4 | 0.9 |
| 32 | F | 49 | 2.6 | NA | 4 | 99 | 1.4 | 0.8 |
| 33 | M | 61 | 2.2 | NA | 6.3 | 119.3 | 1.8 | 1.1 |
| 34 | F | 32 | 3.4 | NA | 4.3 | 76 | 1.1 | 1.1 |
| 35 | M | 61 | 2.9 | NA | 13 | 87.6 | 1.3 | 1.9 |
| 36 | M | 43 | 3.4 | NA | 3.8 | 29.8 | 3.4 | 1.1 |
| Mean±SD |  | 48±12.7 | 2.9±0.6 | 6.6±3.4 | 6.6±3.5† | 46.8±43.2 | 1.7±1† | 1.1±0.3† |
| Median (IQ) |  |  |  |  | 6 (4.1-8) |  | 1.6 (1.1-2.3) | 1.1 (1-1.3) |

| **Table 2B. FSGS patients in remission (n=16)** | | | | | | | |
| --- | --- | --- | --- | --- | --- | --- | --- |
| **Patient** | **Gender** | **Age (years)** | **Serum albumin (g/dl)** | **UPC** | **Urinary Angptl4**  **(ng/g creatinine)** | **Serum Angptl4**  **(ng/ml)** | **Serum creatinine**  **(mg/dl)** |
| 37 | M | 23 | 3.9 | 1.9 | 6 | 0.9 | 1.7 |
| 38 | F | 49 | 2.6 | 1.4 | 1.5 | 2 | 0.8 |
| 39 | M | 61 | 4.3 | 1.7 | 2.6 | 15.3 | 1.1 |
| 40 | M | 65 | 4 | 1.8 | 9.2 | 0.8 | 1 |
| 41 | M | 41 | 4.1 | 0.1 | 3.9 | 5.7 | 1.7 |
| 42 | M | 49 | 3.5 | 0.9 | 10.4 | 1.3 | 1.9 |
| 43 | F | 50 | 3.9 | 0.7 | 3.2 | 26.6 | 0.6 |
| 44 | M | 41 | 4.1 | 0.1 | 3.3 | 1.5 | 1.4 |
| 45 | M | 40 | 4 | 0.1 | 0.3 | 3.1 | 1.8 |
| 46 | M | 39 | 4.3 | 0.2 | 1.3 | 1.1 | 1.4 |
| 47 | M | 57 | 4.1 | 0.8 | 1 | 0.5 | 1.2 |
| 48 | M | 60 | 4.1 | 0.04 | 0 | 6.3 | 1.2 |
| 49 | F | 32 | 3.4 | 0.2 | 3.6 | 1.2 | 1.1 |
| 50 | F | 31 | 4 | 0.1 | 1.7 | 0.4 | 3.7 |
| 51 | M | 32 | 3.7 | 0.8 | 5.4 | 5.4 | NA |
| 52 | M | 36 | 3.9 | 0.8 | 6.1 | NA | 1.1 |
| Mean±SD |  | 44.1±12.2 | 3.8±0.4† | 0.7±0.6 | 3.7±3 | 4.8±7.1† | 1.4±0.7 |
| Median(IQ) |  |  | 4 (2.6-4.1) |  |  | 1.5 (0.9-5.7) | 1.2 (1.1-1.7) |
