# Supplementary material for: Angiopoietin-like-4 and minimal change disease
Source: PLoS One. 2017 Apr 25;12(4):e0176198. doi: 10.1371/journal.pone.0176198 (PMC5404758; doi:10.1371/journal.pone.0176198)
Supplement: S3 Table — MN membranous nephropathy, UPC urine protein to creatinine ratio, Angptl4 angiopoietin-like-4, F female, M male, NA not available, SD standard deviations, IQ interquartile 25–75% percentile, data presented as mean±SD and median (IQ) when data were not normally distributed, † non normally distributed data. (DOC) [file pone.0176198.s008.doc]

**S3 Table.**

| **Table 3a. MN patients in relapse (n=36)** | | | | | | | | |
| --- | --- | --- | --- | --- | --- | --- | --- | --- |
| **Patient** | **Gender** | **Age (years)** | **Serum albumin (g/dl)** | **Proteinuria** | | **Urinary Angptl4**  **(ng/g creatinine)** | **Serum Angptl4**  **(ng/ml)** | **Serum creatinine**  **(mg/dl)** |
|  |  |  |  | **UPC** | **Grams/24 h** |  |  |  |
| 1 | M | 67 | 1.9 | 6.4 | NA | 16.3 | 1.4 | 2 |
| 2 | M | 29 | 2.9 | 10.9 | NA | 20 | 0.7 | 0.5 |
| 3 | M | 63 | 2.5 | 3.8 | NA | 6.9 | 0.3 | 1.2 |
| 4 | M | 40 | 1.9 | 5 | NA | 5.8 | 0.3 | 0.8 |
| 5 | F | 45 | 2.4 | 18.9 | NA | 12.2 | 0.6 | 0.8 |
| 6 | M | 76 | 2.2 | 3.7 | NA | 3.3 | 0.8 | 0.9 |
| 7 | M | 69 | 2.3 | 10.9 | NA | 9.7 | NA | 0.8 |
| 8 | F | 41 | 1.7 | 10.9 | NA | 57.2 | NA | 0.9 |
| 9 | F | 68 | 2.1 | 7.9 | NA | 5.9 | NA | 0.8 |
| 10 | M | 59 | 2.4 | 9.3 | NA | 25.6 | 2.4 | 1.5 |
| 11 | F | 35 | 2.1 | 3 | NA | 1.7 | 0.2 | 0.8 |
| 12 | M | 38 | 2.3 | 2.2 | NA | 1.2 | NA | 1 |
| 13 | M | 67 | 1.9 | 2.3 | NA | 3.4 | 0.6 | 1.1 |
| 14 | F | 49 | 3.2 | 2.6 | NA | 6.4 | 6.1 | 0.8 |
| 15 | M | 72 | 3 | 2.7 | NA | 12.5 | 0.4 | 1.5 |
| 16 | F | 43 | 3.3 | 2.4 | NA | 0.2 | 0.9 | 1 |
| 17 | M | 46 | 2.2 | NA | 7.8 | 8.2 | 1 | 0.9 |
| 18 | M | 56 | 2.1 | NA | 11 | 45.1 | 0.6 | 0.8 |
| 19 | F | 34 | 2.4 | NA | 5.5 | 33.5 | 0.2 | 0.9 |
| 20 | M | 71 | 2.5 | NA | 6.6 | 25 | 1.9 | 1.4 |
| 21 | F | 48 | 2.6 | NA | 5 | 15 | 3.1 | 0.8 |
| 22 | F | 65 | 2.7 | NA | 6.1 | 23.8 | 2 | 1.1 |
| 23 | M | 46 | 2.7 | NA | 3.8 | 34.8 | 3.6 | 1.4 |
| 24 | M | 67 | 1.9 | NA | 12 | 25.2 | 4.6 | 2 |
| 25 | F | 49 | 3.2 | NA | 4.7 | 44.6 | 3.1 | 0.8 |
| 26 | M | 72 | 3 | NA | 5.9 | 19.9 | 2.6 | 1.5 |
| 27 | F | 46 | 3.5 | NA | 3.7 | 31.7 | 1.7 | 1.2 |
| 28 | M | 49 | 3.1 | NA | 6.5 | 12.9 | 2.6 | 0.9 |
| 29 | M | 59 | 2.4 | NA | 5.4 | 24.3 | 2.7 | 1.3 |
| 30 | M | 71 | 3.2 | NA | 4.8 | 23.3 | 1.8 | 1.2 |
| 31 | M | 37 | 3.4 | NA | 7 | 16.5 | 2.4 | 0.9 |
| 32 | F | 57 | 2.7 | NA | 9.4 | 20 | 2.9 | 1.5 |
| 33 | M | 62 | 2.1 | NA | 6.5 | 41.7 | 3.7 | 0.9 |
| 34 | M | 63 | 2.3 | NA | 15 | 18.1 | 2.3 | 0.9 |
| 35 | F | 48 | 1.8 | NA | 13 | 26.7 | 1.5 | 0.7 |
| 36 | M | 41 | 2.1 | NA | 10 | 38.9 | 2.1 | 0.7 |
| Mean±SD |  | 54.1±13† | 2.5±0.5 | 6.4±4.7† | 7.4±3.1 | 19.9±14.1 | 1.9±1.3† | 1±0.3† |
| Median(IQ) |  | 52 (43-67) |  | 4.4 (2.6-10.5) |  |  | 1.8 (0.6-2.6) | 0.9 (0.8-1.2) |

| **Table 3b. MN patients in remission (n=16)** | | | | | | | |
| --- | --- | --- | --- | --- | --- | --- | --- |
| **Patient** | **Gender** | **Age (years)** | **Serum albumin (g/dl)** | **UPC** | **Urinary Angptl4**  **(ng/g creatinine)** | **Serum Angptl4**  **(ng/ml)** | **Serum creatinine**  **(mg/dl)** |
| 37 | M | 45 | NA | 1.87 | 2.5 | NA | NA |
| 38 | M | 59 | NA | 1.89 | 1.9 | 0.4 | 1.38 |
| 39 | F | 57 | NA | 1.66 | 11 | 2.6 | 1.5 |
| 40 | M | 41 | NA | 1.45 | 4.5 | 0.5 | 0.76 |
| 41 | F | 45 | NA | 1.72 | 0.5 | 0.2 | 0.71 |
| 42 | M | 46 | NA | 1.28 | 1.9 | 3.2 | 0.9 |
| 43 | F | 48 | NA | 0.04 | 1.5 | 0.5 | 0.87 |
| 44 | F | 65 | NA | 0.90 | 2.2 | 0.5 | 1.1 |
| 45 | M | 46 | NA | 0.43 | 1.2 | 21 | 1.4 |
| 46 | F | 46 | 3.5 | 0.36 | 0.7 | 1.5 | 1.2 |
| 47 | M | 49 | 3.1 | 0.04 | 1.7 | 0.5 | 0.9 |
| 48 | M | 37 | 3.4 | 0.10 | 0.5 | 1 | 0.97 |
| 49 | M | 62 | NA | 0.69 | 12.2 | 0.6 | 0.94 |
| 50 | M | 63 | NA | 0.81 | 4.5 | 5.6 | 0.9 |
| 51 | F | 48 | NA | 0.30 | 74.7 | 29 | 0.78 |
| 52 | M | 66 | NA | 0.08 | 2 | 1 | 1.1 |
| Mean±SD |  | 51.4±9.1 | 3.3±0.2† | 0.8±0.6 | 7.7±18.2† | 4.5±8.5† | 1±0.2 |
| Median (IQ) |  |  | 3.4 (3.1-3.5) |  | 1.9 (1.2-4.5) | 1 (0.5-3.2) |  |
