# Supplementary material for: Angiopoietin-like-4 and minimal change disease
Source: PLoS One. 2017 Apr 25;12(4):e0176198. doi: 10.1371/journal.pone.0176198 (PMC5404758; doi:10.1371/journal.pone.0176198)
Supplement: S4 Table — UPC urine protein to creatinine ratio, Angptl4 angiopoietin-like-4, F female, M male, NA not available, SD standard deviations, IQ interquartile 25–75% percentile, negative refers to negative protein by dipstick, data presented as mean±SD and median (IQ) when data were not normally distributed, † non normally distributed data. (DOC) [file pone.0176198.s009.doc]

**S4 table**.

| **Characteristics of control subjects (n=18 children)** | | | | | | | |
| --- | --- | --- | --- | --- | --- | --- | --- |
| **Patient** | **Gender** | **Age (years)** | **Serum albumin (g/dl)** | **UPC** | **Urinary Angptl4**  **(ng/g creatinine)** | **Serum Angptl4**  **(ng/ml)** | **Serum creatinine**  **(mg/dl)** |
| 1 | M | 17 | NA | Negative | 0 | NA | NA |
| 2 | M | 13 | NA | Negative | 0 | NA | 0.5 |
| 3 | M | 11 | NA | Negative | 0 | NA | 0.5 |
| 4 | M | 15 | NA | Negative | 0 | NA | 0.4 |
| 5 | M | 17 | NA | Negative | 1 | NA | NA |
| 6 | M | 9 | NA | Negative | 1.25 | NA | NA |
| 7 | F | 5 | NA | Negative | NA | 9.8 | 0.3 |
| 8 | M | 8 | NA | Negative | NA | 1.1 | 0.3 |
| 9 | F | 19 | NA | 0.05 | 17.7 | 0.9 | 0.6 |
| 10 | M | 11 | 4.5 | 0.09 | 2.3 | 1.8 | 0.7 |
| 11 | M | 12 | NA | Negative | 7.3 | 22.2 | NA |
| 12 | F | 9 | NA | 0.09 | 1.3 | NA | NA |
| 13 | F | 17 | 4.8 | 0.07 | 3.5 | 0.5 | 0.7 |
| 14 | F | 19 | 4.7 | 0.04 | 1 | 1.5 | 0.8 |
| 15 | F | 15 | 5.2 | 0.15 | 5.9 | 2 | 0.9 |
| 16 | M | 15 | 5.1 | 0.04 | 3.8 | 4.7 | 0.8 |
| 17 | M | 13 | 4.5 | 0.06 | 5.1 | 4.7 | 0.7 |
| 18 | M | 16 | 4.7 | 0.03 | 3.3 | NA | 0.8 |
| Mean±SD |  | 13.3±3.9 | 4.7±0.2† | 0.06±0.03 | 3.3±4.4† | 4.9±6.6 | 0.6±1.9 |
| Median±IQ |  |  | 4.7 (4.5-5.1) |  | 1.8 (0.2-4.7) | 1.9 (1-5.9) |  |
