# Supplementary material for: Angiopoietin-like-4 and minimal change disease
Source: PLoS One. 2017 Apr 25;12(4):e0176198. doi: 10.1371/journal.pone.0176198 (PMC5404758; doi:10.1371/journal.pone.0176198)
Supplement: S5 Table — Angptl4 angiopoietin-like-4, MCD minimal change disease, FSGS focal segmental glomerulosclerosis, MPGN membranoproliferative glomerulonephritis, * individuals not included in any of the studied groups. Data from these individuals were not included in Table 1 nor figures, UPC urine protein to creatinine ratio, ** Negative refers to negative protein by dipstick. (DOC) [file pone.0176198.s010.doc]

**S5 Table.**

| **Disease** | **Patient number** | **Age (years)** | **Serum albumin (g/dl)** | **UPC (mg/mg)** |
| --- | --- | --- | --- | --- |
| **MCD relapse** | 1 | 7 | 2.5 | 13.7 |
|  | 4 | 10 | 1.9 | 4.2 |
|  | 13 | 8 | 3.9 | 1.1 |
|  | * | 5 | 1.8 | 27.6 |
| **MCD remission** | 51 | 4 | NA | 0.08 |
|  | * | 19 | 4.5 | Negative** |
| **Control** | * | 9 | NA | Negative** |
| **Recurrent FSGS** | * | 11 | 2.2 | 10.8 |
|  | * | 12 | 3 | 2.6 |
|  | * | 8 | 1.7 | 44.1 |
| **FSGS relapse** | * | 3 | 1.7 | 4.8 |
|  | * | 13 | 3.6 | 2.6 |
|  | * | 16 | 2.1 | 15.4 |
| **Lupus nephritis** |  |  |  |  |
| **Class IV** | * | 11 | 3.4 | 1.2 |
| **Class II** | * | 11 | NA | 0.3 |
|  | * | 12 | 3.2 | Negative** |
| **MPGN relapse** | * | 7 | 2.6 | 9 |
| **MPGN remission** | * | 21 | 4.5 | 0.5 |
