# Supplementary material for: Angiopoietin-like-4 and minimal change disease
Source: PLoS One. 2017 Apr 25;12(4):e0176198. doi: 10.1371/journal.pone.0176198 (PMC5404758; doi:10.1371/journal.pone.0176198)
Supplement: S6 Table — Angptl4 angiopoietin-like-4, MW molecular weight, kDa kilodalton, aa aminoacids. (DOC) [file pone.0176198.s011.doc]

**S6 Table.**

| **Author**  **Company/Catalog** | **Type of Tissue** | **Type of antibody** | **Source** | **Antibody raised against** | **MW (kDa)** | **Secondary antibody** |
| --- | --- | --- | --- | --- | --- | --- |
| Present study  Santa Cruz/sc-66806 | Frozen | Polyclonal | Rabbit | aa 51-250  (Near N terminus) | ~50 | Chicken anti-rabbit |
| Chugh’s group^10^  Customized | Not provided | Polyclonal | Rabbit | N-term-1-374aa | ~50 | Not provided |
| Li ^13^, Peng^15^  Santa Cruz/ sc-34113 | Frozen | Polyclonal | Goat | Near N terminus | ~50 | Donkey anti-goat |
