# Supplementary material for: Angiopoietin-like-4 and minimal change disease
Source: PLoS One. 2017 Apr 25;12(4):e0176198. doi: 10.1371/journal.pone.0176198 (PMC5404758; doi:10.1371/journal.pone.0176198)
Supplement: S7 Table — Angptl4 angiopoietin-like-4, aa aminoacids, y year-old, N refers to the number of patients, SD standard deviation, SEM standard error of mean, IQ interquartile, * “Elisa assay virtually identical to the Duoset Elisa Angptl4 offered commercially by R&D (DY3485)”. (DOC) [file pone.0176198.s012.doc]

**S7 Table**.

| **Author** | **Company** | **Type of antibody** | **Antibody raised against** | **Population** | **Plasma (p) or serum (s) Angptl-4** | **Fasting** |
| --- | --- | --- | --- | --- | --- | --- |
| **Chugh’s group^5^** | R&D/Duoset Elisa, DY3485 | Polyclonal, raised in goat | aa 26-406 | N 19 healthy controls  Mean age 31.5 y | Mean±SD 59.6 ± 22.1 ng/ml (p)  Range 24.78-119.28 ng/ml | Yes |
| **Present**  **Study** | Sigma/ RAB0017 | Polyclonal, raised in goat | aa 26-406 | N 10 healthy controls  Mean age 13.4 y | Mean±SD 4.9 ± 6.6 ng/ml (s)  Range 0.5-22.2 ng/ml | No |
| **Kersten^13^*** | R&D AF3485 (capture)  BAF 3485 (detection)* | Polyclonal, raised in goat | aa 26-406 | N 25 mildly hyperlipidemic subjects | Range ~0 to ~37 ng/ml (most samples were below<10 ng/ml) (p) | Overnight |
| **Van der Kolk^14^** | R&D AF3485 (capture)  BAF 3485 (detection) | Polyclonal, raised in goat | aa 26-406 | N 150 overweight and obese pre-diabetic humans | Mean ± SEM 5.7 ± 0.2 ng/ml (p) | Overnight |
| **Tjeerdema^15^** | R&D AF3485 (capture)  BAF 3485 (detection) | Polyclonal, raised in goat | aa 26-406 | N 90 healthy controls  Mean age 56.5 y | Median 3.7 ng/ml (p)  IQ range (2.8-5.2) | Unknown |
| **Jonker^16^** | R&D AF3485 (capture)  BAF 3485 (detection) | Polyclonal, raised in goat | aa 26-406 | 3 healthy control groups  A: N 22  B: N 10  C: N 15 | Baseline Median (IQ): (p)  A: 10.6 ng/ml (7.6-17.6 ng/ml)  B: 13.2 ng/ml (8.1/24.2 ng/ml)  C: 13.9 ng/ml (8.2-22 ng/ml) | No at baseline |
| **Robciuc^17^** | R&D/Duoset Elisa/DY3485  Home made | Polyclonal, raised in goat | aa 26-406 | Subpopulation from Health Examination Survey  N 125 men, mean age 55.2 y  N 125 women, mean age 54.9 y | Mean±SD for men 20.7±25.7 ng/ml (µg/l)  and for women 16.3±20.6 ng/ml (µg/l) (s) | Unknown |
